# Independent Risk Factors and Combined Predictive Model for Severe *Mycoplasma pneumoniae* Pneumonia in Children: The Role of Vitamin D Deficiency

**DOI:** 10.3390/children13101444

**Published:** 2026-10-21

**Authors:** Mengting Zhang, Yu Fu, Ruimeng Ma, Zonglang Yu, Shuping Jin, Xiaoying Zhang

**Affiliations:** Shanghai Ninth People’s Hospital, School of Medicine, Shanghai Jiao Tong University, Shanghai 200011, China; zmtaaaaa@sjtu.edu.cn (M.Z.); fuyu18856030890@sjtu.edu.cn (Y.F.); sakura666@sjtu.edu.cn (R.M.); yuzonglang@sjtu.edu.cn (Z.Y.); 330483200108230066@sjtu.edu.cn (S.J.)

**Keywords:** 25-hydroxyvitamin D, severe *Mycoplasma pneumoniae* pneumonia, pulmonary function, children

## Abstract

**Highlights:**

**What are the main findings?**

- Severe *Mycoplasma pneumoniae* pneumonia (SMPP) in children was shown to be independently associated with serum 25-hydroxyvitamin D [25(OH)D] deficiency, increased C-reactive protein (CRP), raised lactate dehydrogenase (LDH), prolonged fever duration, and the presence of pleural effusion. Importantly, vitamin D deficiency was associated with higher odds of SMPP and may serve as an underrecognized risk marker in children with MPP.
- The predictive model integrating all five risk variables achieved an AUC of 0.866 for identifying SMPP, which outperformed any single indicator, with a sensitivity of 88.5% and specificity of 74.0%.

**What are the implications of the main findings?**

- All variables included in the model are routinely measurable in standard pediatric clinical settings. This risk stratification tool may help recognize children with SMPP at higher risk, though external validation is needed to verify its performance and clinical utility.
- The correlation between vitamin D deficiency and SMPP suggests a possible link between immune nutritional status and progression of pediatric MPP, warranting further investigation of vitamin D supplementation as a potential adjunctive strategy.

**Abstract:**

**Objectives**: Vitamin D deficiency had been associated with the pathophysiology of respiratory infections. This research sought to assess the correlation between disease severity, serum 25-hydroxyvitamin D [25(OH)D] and lung function in children with *Mycoplasma pneumoniae* pneumonia (MPP). **Methods**: A cohort of 102 children with MPP aged 6–17 years was recruited. All enrolled participants were 6–14 years old, and they were categorised into a mild group (*n* = 50) and a severe group (*n* = 52). Between-group comparisons were performed for demographic characteristics, serum 25(OH)D concentrations, laboratory indicators, imaging results, and pulmonary function. Logistic regression was employed to determine risk variables, and their predictive ability for SMPP was examined by ROC. **Results**: The severe group had markedly reduced serum 25(OH)D concentrations compared to the mild group (17.23 ± 6.81 vs. 20.49 ± 6.09 ng/mL; *p* = 0.013). Fever duration was prolonged, and CRP, LDH, D-dimer, NLR, AST and IL-6 were elevated in the severe group (all *p* < 0.05). Moreover, pleural effusion and pulmonary consolidation were more commonly observed in the severe group (*p* < 0.05). Pulmonary function showed no disparities between groups. Logistic regression identified 25(OH)D < 12 ng/mL (OR = 11.31), elevated CRP (OR = 1.033), elevated LDH (OR = 1.009), prolonged fever duration (OR = 1.552), and pleural effusion (OR = 41.86) as independent risk variables for SMPP. The integrated model produced an AUC of 0.866 (95% CI: 0.795–0.937), demonstrating 88.5% sensitivity and 74.0% specificity. **Conclusions**: 25(OH)D < 12 ng/mL, elevated CRP and LDH, prolonged fever duration, and pleural effusion are associated with severe pneumonia in children with MPP aged 6–14 years. Their combined use has exploratory predictive potential for identifying severe disease in children with MPP.

## 1. Introduction

*Mycoplasma pneumoniae* pneumonia (MPP) represents the predominant form of community-acquired pneumonia (CAP) in children aged 5 and above [1]. The percentage of severe *Mycoplasma pneumoniae* pneumonia (SMPP) cases has risen globally over recent years [2]. SMPP can lead to serious intrapulmonary and extrapulmonary complications, increasing the risk of mortality in affected children [3]. The clinical manifestations of SMPP are generally non-specific during the initial infection stage, making it essential to promptly identify high-risk patients to enhance clinical decision-making and long-term prognosis.

Beyond its established role in regulating calcium–phosphate balance and preserving skeletal health, vitamin D also exerts broad immunomodulatory effects on the respiratory mucosa, modulating both innate and adaptive immune responses against pathogens [4]. Accumulating epidemiological evidence associates a deficit in vitamin D with heightened vulnerability to respiratory tract infections and indicates that low vitamin D status may be linked to greater disease severity [5]. But findings across pediatric studies remain inconsistent, and the value of vitamin D status as a severity biomarker in MPP remains unclear.

Children with MPP may develop varying degrees of pulmonary function impairment, which can be persistent or even irreversible in severe cases [6]. However, few investigations have systematically explored the correlations among serum 25-hydroxyvitamin D [25(OH)D], disease severity, and pulmonary function in children with MPP, nor assessed whether 25(OH)D combined with routine markers can enhance early prediction of severe disease.

Therefore, the study was designed to characterize and compare clinical manifestations, laboratory indices (including serum 25(OH)D), imaging features, and pulmonary function patterns in children with SMPP versus mild MPP. We additionally evaluated the utility of these variables for predicting severe disease, with the goal of supporting early clinical recognition and risk stratification of SMPP in children.

## 2. Materials and Methods

### 2.1. Patient Population

This research obtained ethical clearance from the Medical Ethics Committee of Shanghai Ninth People’s Hospital (Affiliated to Shanghai Jiao Tong University School of Medicine), with approval reference SH9H-2024-T331-1.

We enrolled a total of 102 pediatric inpatients with a confirmed diagnosis of MPP who received inpatient care in the Department of Pediatrics during the period from July 2023 to July 2024.

The enrollment criteria were as follows: (1) between 6 and 17 years of age; (2) conforming to the diagnostic standards for MPP outlined in the 2023 edition of the Guidelines for the Diagnosis and Treatment of *Mycoplasma pneumoniae* Pneumonia in Children [7].

The exclusion criteria were as follows: (1) concomitant bacterial, viral, or fungal respiratory infections; (2) comorbidities including hepatic or renal dysfunction, asthma, diabetes mellitus, thyroid disorders, autoimmune diseases, and congenital heart disease; (3) a history of long-term immunotherapy; (4) incomplete clinical data.

We set the minimum age at 6 years to ensure that participants can achieve the required performance levels on the pulmonary function tests. Based on the severity classification criteria specified in Section 2.2, all enrolled patients were stratified into two cohorts: mild MPP (MMPP) and severe MPP (SMPP).

### 2.2. Severity Classification

Patients were categorized as having SMPP if they presented with any of the following clinical and radiological features: deteriorated general condition, refusal to eat or evidence of dehydration, altered level of consciousness, significant tachypnea (respiratory rate > 70 breaths/min in infants and >50 breaths/min in older children) or cyanosis with a peripheral oxygen saturation (SpO_2_) of ≤92%, dyspnea with grunting respiration, nasal flaring, or intercostal retractions; radiologically, single-lobe involvement of ≥2/3 with dense consolidation, or consolidation in ≥2 lobes (which may be accompanied by moderate-to-large pleural effusion), diffuse unilateral or bilateral involvement of ≥4/5 lung fields with bronchiolitis, atelectasis, pulmonary necrosis or lung abscess, and other intrapulmonary or extrapulmonary complications. Those who failed to satisfy the above criteria were categorised as the MMPP group.

### 2.3. Sample-Size Calculation

PASS 15 was used to estimate sample size. The Two-Sample T-Tests Assuming Equal Variance procedure was adopted. The type I error (α) was set at 0.05 (two-sided), and the type II error (β) was set at 0.20 (one-sided). Based on previously published data in children with MPP [8], we assumed mean serum 25(OH)D concentrations of 23.08 ng/mL for the SMPP group and 32.07 ng/mL for the MMPP group, with standard deviations of 11.0 ng/mL and 19.2 ng/mL, respectively. The study was designed with a 1:1 allocation ratio (k = 1). The calculated effect size (Cohen’s d) was 0.57. Without accounting for loss to follow-up, the required sample size was 48 participants per group. After incorporating an anticipated exclusion rate of 20%, the target sample size before screening was 60 participants per group (120 in total). A total of 120 patients were initially screened, and 102 eligible children were finally included after exclusion according to prespecified criteria.

### 2.4. Data Collection

#### 2.4.1. Clinical and Laboratory Parameters

Demographic and clinical features gathered for examination were age, gender, body mass index (BMI), duration of fever, peak temperature, hospital stay duration, and presence of wheezing, dyspnea, or chest pain.

Fasting venous blood was collected for routine laboratory assessment within 24 h after hospital admission. The following parameters were evaluated: white blood cell count (WBC), C-reactive protein (CRP), lactate dehydrogenase (LDH), neutrophil-to-lymphocyte ratio (NLR), erythrocyte sedimentation rate (ESR), D-dimer, ferritin, alanine aminotransferase (ALT), aspartate aminotransferase (AST), and cytokine levels (IL-1β, IL-4, IL-6, IL-17, IFN-γ, TNF-α).

We quantified serum 25(OH)D levels using a standardized protocol as follows. First, 2 mL of peripheral venous blood was collected at admission into a plain biochemistry tube and centrifuged at 3500 r/min for 10 min (centrifugation radius: 157 mm). The supernatant was then aspirated into a clean centrifuge tube for downstream analysis. Vitamin D status was determined based on total serum 25(OH)D concentrations, following the recommendations set out in the 2022 European Micronutrient Guidelines. We employed liquid chromatography-tandem mass spectrometry (LC-MS/MS, 3200MD, AB Sciex, Framingham, MA, USA) for detection, which is universally acknowledged as the reference standard method for measuring circulating 25(OH)D, with superior specificity and sensitivity and minimal interference from other compounds. All measurements were performed strictly in accordance with the manufacturer’s instructions.

The status of serum 25(OH)D was divided into three strata according to the guidelines set out by the Institute of Medicine (IOM) and the Chinese national standard for vitamin D screening: deficiency (<12 ng/mL), insufficiency (12–20 ng/mL), and adequacy (>20 ng/mL) [9,10].

#### 2.4.2. Chest Imaging

All patients had a chest radiography or CT examination upon admission. Imaging findings, including lesion distribution, extent of infiltration, presence of pleural effusion, atelectasis, and consolidation, were recorded.

#### 2.4.3. Pulmonary Function Tests

A MasterScreen Pediatric Pulmonary Function Analyzer (Jaeger, Würzburg, Germany) was used to perform pulmonary function tests within 1–3 days of admission. All tests were conducted by certified technicians specialized in pediatric pulmonary function assessment, in strict accordance with the standard operating procedures recommended by the American Thoracic Society (ATS) [11].

Test parameters: Recorded parameters included forced vital capacity (FVC), forced expiratory volume in 1 s (FEV_1_), FEV_1_/FVC ratio, and forced expiratory flows at 25%, 50%, and 75% of FVC (FEF_25_, FEF_50_, FEF_75_). Spirometric parameters were reported as percentages of predicted values derived by the pulmonary function device using the pediatric reference equations for Chinese children aged 4–18 years established by Wu et al. (2025), with age, sex, and height taken into consideration [12].

Pre-test precautions: Drugs affecting airway function were discontinued for at least 12 h before testing, including β_2_-receptor agonists, inhaled corticosteroids, muscarinic receptor antagonists, antihistamines, and leukotriene receptor antagonists. Tests were performed 1–2 h after a meal to avoid discomfort during forced breathing maneuvers.

For pulmonary function assessment, test results were sorted into three categories following the interpretive criteria specified in the Guidelines for Pediatric Pulmonary Function (2): Lung Volume and Ventilatory Function, published by the Respiratory Group of the Pediatrics Society, Chinese Medical Association.

- Normal pulmonary function (NPF): FVC, FEV_1_, FEV_1_/FVC, FEF_50_, and FEF_75_ all ≥80% of predicted values;
- Small airway dysfunction (SAD): Ventilatory parameters (FVC, FEV_1_, FEV_1_/FVC) within the normal range, but at least two of FEF_50_, FEF_75_, or maximum mid-expiratory flow (MMEF) are <65% of predicted values, indicating isolated small airway impairment;
- Abnormal ventilatory function (VD): FEV_1_/FVC < 80% of predicted values, with or without reduced FVC.

### 2.5. Statistical Analysis

SPSS Statistics version 29.0 was used for the statistical analyzes. Continuous variables were summarized according to normality status: normally distributed data were presented as mean ± standard deviation (SD), whereas non-normal data were described using median (interquartile range, IQR). Independent samples t-tests were used for between-group comparisons of normally distributed continuous outcomes; the Mann–Whitney U test was used for variables deviating from a normal distribution. Categorical clinical data were summarized as counts and corresponding percentages [*n* (%)], with inter-group disparities assessed via the chi-square or Fisher’s exact test where suitable. A multivariate logistic regression model was developed to ascertain independent risk variables for SMPP, with the occurrence of SMPP serving as the dependent variable. Variables with *p* < 0.1 in univariate analysis were entered into the model using the forward stepwise likelihood ratio method. Univariate analysis showed that fever peak, fever duration, CRP, NLR, LDH, AST, D-dimer, IL-6, pleural effusion, lung consolidation, and serum 25(OH)D level were linked to MPP severity. For univariate analysis, serum 25(OH)D was categorized into three strata: <12 ng/mL, 12–20 ng/mL, and >20 ng/mL to explore the trend in the proportion of SMPP across different vitamin D levels. In the multivariate regression model, 25(OH)D was dichotomized as <12 ng/mL versus ≥12 ng/mL to evaluate the independent effect of vitamin D deficiency. Receiver operating characteristic (ROC) curve analysis was implemented to quantify the predictive performance of relevant variables; corresponding area under the curve (AUC) values, diagnostic sensitivity, and specificity were subsequently obtained. All statistical tests were two-sided, and a *p* value < 0.05 was established as the cutoff point for statistical significance.

## 3. Results

### 3.1. Baseline Characteristics

A total of 102 children were enrolled in this cohort, with ages ranging from 6 to 14 years. No notable differences in age, sex, BMI, hospitalization duration, the prevalence of pulmonary rales, tachypnea, or hypoxaemia were observed between the MMPP and SMPP groups (all *p* > 0.05). In contrast to the MMPP group, children in the SMPP group had a significantly higher fever peak and longer fever duration (all *p* < 0.05; Table 1).

### 3.2. Laboratory and Imaging Findings

Compared with the MMPP group, children in the SMPP group had markedly elevated serum levels of CRP, NLR, LDH, D-dimer, AST, IL-6, and IFN-γ (all *p* < 0.05). No notable changes between groups were seen in WBC, ferritin, ALT, ESR, IL-1β, IL-4, IL-17, or TNF-α (all *p* > 0.05). Regarding the chest imaging results, the SMPP group had a greater percentage of lung consolidation and pleural effusion than the MMPP group (both *p* < 0.05; Table 2).

### 3.3. Serum 25(OH)D Levels

**Figure 1:**
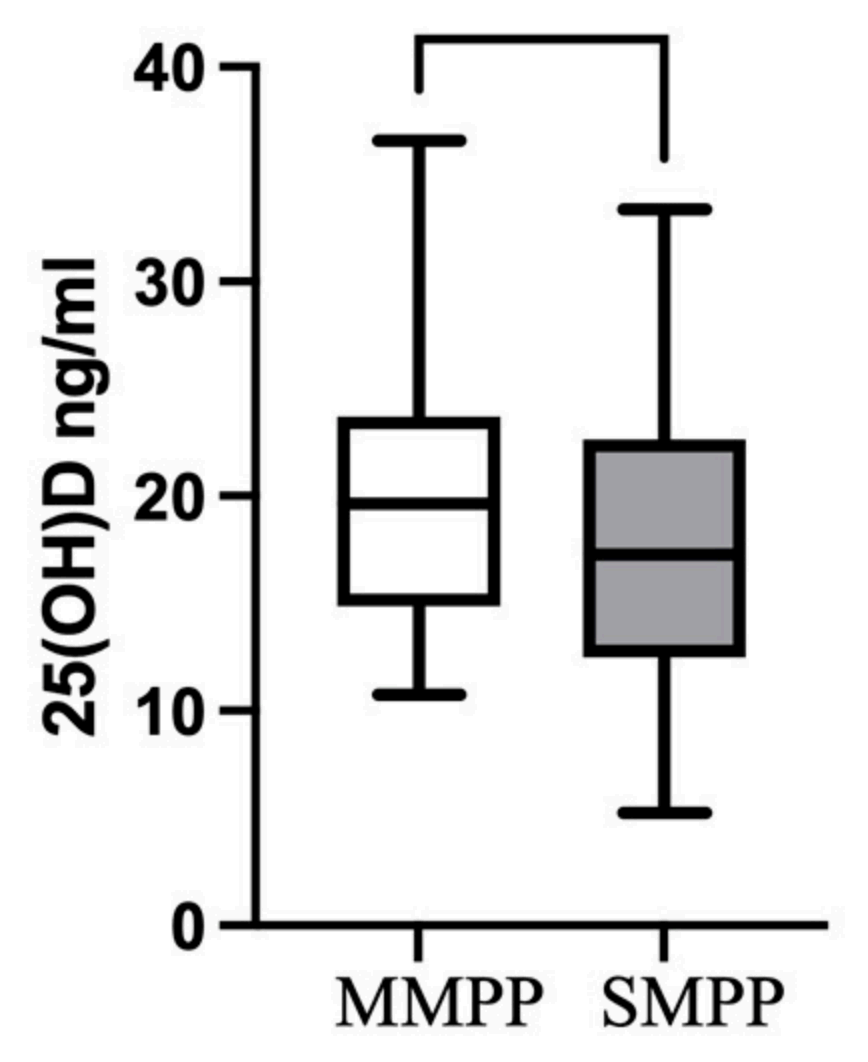
Comparison of serum 25(OH)D levels between the MMPP and SMPP groups. Data are presented as box-and-whisker plots showing median, interquartile range, and range.

The serum 25(OH)D level was significantly lower in the SMPP group compared to the MMPP group (17.23 ± 6.81 vs. 20.49 ± 6.09 ng/mL; *t* = 2.549, *p* = 0.013). When patients were stratified by vitamin D status (deficiency: <12 ng/mL; insufficiency: 12–20 ng/mL; adequacy: >20 ng/mL), the proportion of severe cases was highest in the deficiency group (86.7%), followed by the insufficiency group (50.0%) and the adequacy group (37.8%). The difference in SMPP proportion across the three strata was statistically significant (χ^2^ = 10.221, *p* = 0.006; Figure 1, Table 3).

### 3.4. Comparison of Lung Function Between Mild and Severe Patients

Regarding acute-phase pulmonary function parameters, no notable discrepancies were seen between the SMPP and MMPP groups in FVC, FEV_1_, FEV_1_/FVC, FEF_25_, FEF_50_, or FEF_75_ (all *p* > 0.05). Similarly, there was no notable disparity between groups for the distribution of pulmonary function categories (normal function, small airway dysfunction, and ventilatory dysfunction) (χ^2^ = 0.294, *p* = 0.863; Table 4 and Table 5).

### 3.5. Independent Risk Factors for SMPP

Multivariate analysis demonstrated that 25(OH)D deficiency (<12 ng/mL) (β = 2.426, *p* = 0.017, OR = 11.31, 95% CI: 1.54–83.22), longer fever duration (β = 0.440, *p* = 0.003, OR = 1.552, 95% CI: 1.16–2.07), elevated CRP (β = 0.032, *p* = 0.029, OR = 1.033, 95% CI: 1.00–1.06), elevated LDH (β = 0.009, *p* = 0.049, OR = 1.009, 95% CI: 1.00–1.01), and presence of pleural effusion (β = 3.734, *p* = 0.002, OR = 41.86, 95% CI: 3.82–459.26) were independent risk factors for SMPP (Table 6).

### 3.6. ROC Curve Analysis Predicting SMPP

ROC curve analysis was performed to assess the diagnostic value of each independent risk variable and the combined multivariate model for identifying SMPP.

For individual indicators, CRP had the highest predictive accuracy with an ideal threshold of 9.25 mg/L, yielding an AUC of 0.695 (95% CI: 0.592–0.788, *p* < 0.001), a sensitivity of 82.7%, and a specificity of 54.0%. This was followed by fever duration (optimal cutoff: 5.5 days; AUC = 0.685, 95% CI: 0.582–0.788, *p* < 0.001; sensitivity: 71.2%; specificity: 60.0%), LDH (optimal cutoff: 299.5 U/L; AUC = 0.624, 95% CI: 0.513–0.735, *p* = 0.028; sensitivity: 48.0%; specificity: 90.0%), pleural effusion (AUC = 0.615, 95% CI: 0.506–0.724, *p* = 0.039; sensitivity: 25.0%; specificity: 98.0%), and 25(OH)D deficiency (AUC = 0.605, 95% CI: 0.495–0.715, *p* = 0.061; sensitivity: 25.0%; specificity: 96.0%), which showed borderline statistical significance.

**Figure 2:**
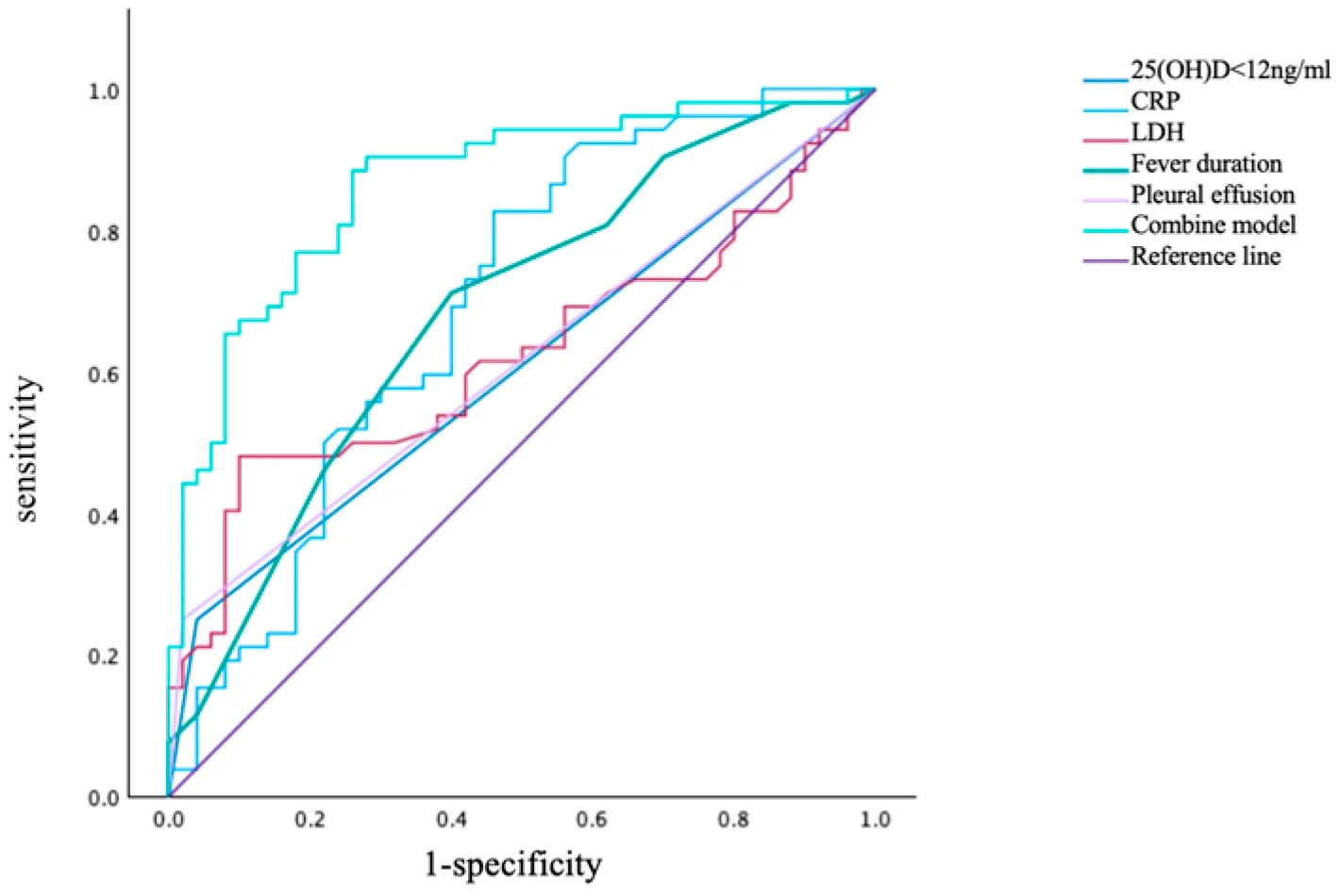
Receiver operating characteristic (ROC) curves for predicting SMPP using individual risk factors and the combined multivariate model.

The combined model derived from multivariate logistic regression (incorporating all five independent risk factors) achieved the best predictive performance, with an AUC of 0.866 (95% CI: 0.795–0.937, *p* < 0.001), a sensitivity of 88.5%, and a specificity of 74.0% (Figure 2).

## 4. Discussion

MPP is prevalent worldwide, with cyclic local outbreaks occurring every 3 to 7 years, and infection rates can exceed 80% during epidemic peaks [13]. According to surveillance data from the Chinese Respiratory Pathogen Surveillance System [14], the incidence of MPP cases among children under 15 in Beijing increased by 92.3% to 94,099 between September and November 2023. Along with the rising incidence of MPP, the proportion of SMPP cases has also increased markedly. Children with SMPP may develop irreversible impairment of pulmonary function or structural lung damage, resulting in chronic cough, recurrent wheezing, and repeated respiratory infections. A proportion of children with SMPP also develop extrapulmonary complications, which pose a significant risk to pediatric health [15].

The early identification of SMPP continues to pose a clinical challenge, and existing data regarding risk factors for SMPP in children remain scarce. In the current investigation, we detected five risk factors for SMPP in pediatric MPP patients: serum 25(OH)D deficiency, increased CRP, heightened LDH, prolonged fever duration, and presence of pleural effusion. Furthermore, the combined predictive model incorporating these five factors exhibited favorable diagnostic performance for SMPP.

### 4.1. Vitamin D Deficiency and SMPP Severity

Vitamin D exerts pleiotropic effects on immune regulation, and accumulating evidence links vitamin D deficiency to heightened severity of respiratory tract infections [4]. Respiratory epithelial cells, alveolar macrophages, B lymphocytes, T lymphocytes, and dendritic cells all express 1α-hydroxylase and vitamin D receptors (VDR) [16]. Mechanistically, vitamin D signaling enhances the secretion of antimicrobial peptides, including cathelicidin and β-defensin, from airway epithelial cells, which strengthens mucosal pathogen clearance and confers protection against pulmonary infection [17]. Moreover, vitamin D inhibits the over-release of pro-inflammatory cytokines, thereby dampening dysregulated inflammatory responses and reducing tissue damage [18].

Vitamin D deficiency is widely recognized as a significant worldwide health concern [19]. During *Mycoplasma pneumoniae* infection, vitamin D deficiency may impair respiratory mucosal defense, facilitating the overgrowth of the pathogen in the respiratory tract. Meanwhile, diminished vitamin D signaling compromises immune regulation, amplifying inflammatory cascades, exacerbating lung parenchymal damage, and ultimately promoting progression to SMPP. Supporting this hypothesis, multiple studies have documented a significant frequency of vitamin D deficiency among children suffering from acute respiratory infections, and lower circulating 25(OH)D levels have been linked to greater disease occurrence and more severe clinical outcomes [20]. For instance, a study of 1016 infants hospitalized for bronchiolitis found that those with 25(OH)D levels <20 ng/mL had higher rates of critical care unit admission and prolonged hospitalizations [21]. A Mendelian randomization study including over 110,000 participants followed for 38 years also found that genetically reduced vitamin D status was associated with an increased susceptibility to bacterial pneumonia [22].

In agreement with existing published evidence, we observed a marked reduction in serum 25(OH)D concentrations in children with SMPP versus the mild MPP group. Notably, vitamin D deficiency was independently associated with SMPP (OR = 11.31) [23]. These findings extend the existing body of data and support the potential utility of vitamin D status evaluation for risk stratification in pediatric MPP patients.

### 4.2. Inflammatory Biomarkers as Predictors of SMPP

CRP is an acute-phase reactant synthesized by hepatocytes that rises rapidly within 4–6 h of inflammatory stimulation or tissue injury. The magnitude of CRP elevation reflects the intensity of infection and systemic inflammation, and is largely independent of age, sex, and anemia status. Previous studies have shown that CRP is markedly elevated in the early stage of SMPP, serving as a useful early predictor and informing decisions regarding glucocorticoid initiation [24]. In our analysis, the SMPP group exhibited significantly higher CRP concentrations, with an ideal diagnostic threshold value of 9.25 mg/L and a corresponding AUC of 0.695. CRP demonstrated high sensitivity (82.7%) but only moderate specificity (54.0%), indicating that it is suitable for early screening but should be interpreted alongside other indicators to improve diagnostic accuracy.

As a key glycolytic enzyme, LDH is ubiquitously present across human tissues. In the setting of pulmonary inflammation, hypoxia, or cellular necrosis, LDH is released from injured cells into the bloodstream, which results in increased serum concentrations [25]. Accumulating evidence has consistently identified LDH as an independent predictor of SMPP. In research by Liu et al., an LDH cutoff value of 412.50 U/L yielded a sensitivity of 62.50% and a specificity of 89.71% for SMPP prediction [26]. Separately, other investigators have suggested combined thresholds of CRP 36.0 mg/L and LDH > 250 U/L for identifying SMPP in children [27]. In our cohort, the optimal LDH cutoff was 299.5 U/L, with a high specificity of 90.0%. The variation in cutoff values across studies may be attributable to differences in patient age, disease stage at sampling, and laboratory assay methods. The high specificity of LDH makes it valuable for confirming SMPP in clinically suspected cases.

### 4.3. Clinical and Radiographic Risk Factors

Prolonged fever is a well-recognized clinical marker of SMPP. In our study, fever duration was significantly longer in children with SMPP, with an optimal cutoff of 5.5 days yielding an AUC of 0.685. This finding is consistent with previous reports [28], and likely reflects sustained pathogen replication and exaggerated systemic inflammation in severe disease. Fever lasting more than 5 days in a child with MPP should therefore prompt close monitoring for progression to severe disease.

Pleural effusion is a common pulmonary complication of SMPP. Consistent with prior research findings, our multivariate analysis validated pleural effusion as an independent risk variable for SMPP [24]. *Mycoplasma pneumoniae* infection induces pleural inflammation and increased vascular permeability, leading to exudative effusion; the presence of pleural effusion accordingly indicates more extensive pulmonary involvement and greater disease severity. In ROC analysis, pleural effusion showed high specificity (98.0%) but relatively low sensitivity, supporting its role as a confirmatory rather than a screening marker.

### 4.4. Pulmonary Function Findings

We observed no statistically significant between-group differences in acute-phase pulmonary function parameters, including FVC, FEV_1_, FEF_25_, FEF_50_, and FEF_75_, nor in the distribution of pulmonary function classifications (normal, isolated small airway dysfunction, ventilatory dysfunction). This result is inconsistent with some previous studies [29]. Several factors may account for this discrepancy. First, all pulmonary function tests in our study were performed within 1–3 days of admission during the acute phase, whereas some prior studies included patients tested during the convalescent phase when functional deficits may be more apparent. Second, we did not perform age-stratified analysis, and pulmonary function reference values vary substantially across pediatric age groups, which may have masked true differences. Third, we did not stratify by wheezing status, and small airway abnormalities are more pronounced in children with wheezing symptoms. Finally, our SMPP group consisted predominantly of moderately severe cases, with a low proportion of extremely severe disease, which may have been insufficient to produce measurable differences in spirometric parameters. Further large-scale studies stratified by age, symptom phenotype, and disease stage are warranted to clarify the trajectory of pulmonary function impairment in pediatric SMPP.

### 4.5. Combined Predictive Model and Clinical Implications

The combined multivariate model incorporating all five independent risk variables achieved an AUC of 0.866, which was substantially higher than that of any single indicator. The combined model also balanced sensitivity (88.5%) and specificity (74.0%), demonstrating favorable overall diagnostic performance in this cohort. These findings indicate that an integrated model incorporating nutritional (vitamin D status), inflammatory (CRP, LDH), clinical (fever duration), and radiographic (pleural effusion) parameters may help improve early identification of SMPP in children in this setting. As a candidate risk stratification tool, it may assist clinicians in detecting high-risk patients who could benefit from surveillance and earlier treatment intensification; nevertheless, it remains unclear where its use would translate to improved clinical outcomes.

Importantly, our study integrates serum 25(OH)D—a nutritional biomarker suitable for routine clinical measurement—into the SMPP prediction framework, which represents a key strength of this work. All variables assessed are routinely available in clinical practice, although further external validation in independent cohorts is required before the model can be considered generalizable or implemented widely in routine care.

### 4.6. Limitations

Taking into account the characteristics of children with MPP, this study used clinically accessible indicators to investigate risk factors for SMPP. It incorporated newly measured serum 25(OH)D into the predictive model, which has certain application and reference values. This research is constrained by many restrictions.

First, fever duration reflects the clinical course after hospital admission, which limits the applicability of this model for early prediction at disease onset. Second, the low events-per-variable ratio, wide confidence intervals, and lack of calibration and internal validation raise concerns of potential overfitting and optimistically biased performance estimates. Third, seasonal variation in serum 25(OH)D and other potential confounders, including vitamin D supplementation and sun exposure, were not adjusted for in the analysis. In addition, the subgroup with vitamin D deficiency was small (*n* = 15), and reverse causation cannot be excluded, given that acute inflammation may lower circulating 25(OH)D levels; therefore, causal inference between vitamin D status and SMPP cannot be drawn from this retrospective analysis. Fourth, pulmonary function was only assessed during the acute phase of illness. Furthermore, since pleural effusion remains one component of the composite SMPP definition, incorporation bias may exist. This bias could overestimate the odds ratio of pleural effusion and inflate the AUC of our predictive model.

Moreover, this was a single-center retrospective study, and the developed prediction model has not been validated in an external independent cohort. In addition, the relatively small sample size may compromise the stability of our findings. Accordingly, future research should adopt multicenter, large-scale prospective designs to validate and refine this predictive model.

## 5. Conclusions

Five independent risk variables for SMPP were found in this group of children with MPP: serum 25(OH)D deficiency, elevated CRP, elevated LDH, prolonged fever duration, and presence of pleural effusion. Notably, vitamin D deficiency was linked to severe illness in pediatric patients with MPP.

The combined multivariate model incorporating these five factors yielded an AUC of 0.866 for predicting SMPP, outperforming any individual indicator. Given that all variables are readily obtainable in routine clinical practice, this exploratory prediction model holds promise as a practical tool for early risk stratification. Nevertheless, given the limitations of our single-center retrospective cohort, its clinical application still requires further external validation before it can guide routine timely clinical intervention to improve outcomes among children with MPP.

No significant between-group differences in acute-phase spirometric parameters were detected in the present study. Our findings should be interpreted cautiously considering our study sample characteristics. Further extensive, multicenter prospective trials are necessary to validate these results.

## Figures and Tables

**Table 1:** Baseline characteristics of the two groups.

Variable	MMPP (*n* = 50)	SMPP (*n* = 52)	Statistic	*p*
Age (years)	8.00 (7.17, 9.60)	8.67 (7.42, 9.81)	−1.021	0.307
Sex (male/female)	27/23	22/30	1.396	0.237
BMI (kg/m^2^)	16.13 ± 3.24	15.70 ± 2.49	0.753	0.453
Fever peak (°C)	39.27 ± 0.62	39.57 ± 0.57	−2.502	0.014
Fever duration (days)	4.90 ± 1.99	6.29 ± 2.09	−3.282	<0.001
Hospital stays (days)	4.74 ± 1.37	5.23 ± 1.50	−1.723	0.088
Rales, *n* (%)	31 (62.0%)	25 (48.1%)	1.425	0.233
SpO_2_ ≤ 92%, *n* (%)	0 (0%)	3 (5.7%)	Fisher	0.132
Tachypnea, *n* (%)	0 (0%)	2 (3.8%)	Fisher	0.326

Note. Data are presented as *n* (%), mean ± SD, or median (P25, P75). Fisher represents Fisher’s exact test.

**Table 2:** Laboratory and imaging findings between the two groups.

Indicator	MMPP (*n* = 50)	SMPP (*n* = 52)	Statistic	*p*
WBC (10^9^/L)	7.65 ± 2.59	7.33 ± 1.93	0.683	0.496
NLR	2.12 ± 1.04	2.88 ± 2.31	−2.123	0.036
CRP (mg/L)	7.95 (1.45, 18.23)	18.35 (10.68, 27.28)	−3.357	<0.001
LDH (U/L)	265.58 ± 45.12	316.90 ± 113.04	−3.032	0.003
Ferritin (ng/mL)	126.2 (99.1–148.8)	132.1 (108.5–198.3)	1075.000	0.132
ESR (mm/h)	27.80 ± 15.47	31.23 ± 13.96	−1.177	0.242
D-dimer (mg/L)	0.18 (0.11, 0.24)	0.22 (0.14, 0.36)	−2.643	0.008
FIB (g/L)	3.40 ± 0.65	3.53 ± 0.64	−1.030	0.306
ALT (U/L)	16.36 ± 8.14	20.08 ± 13.92	−1.654	0.102
AST (U/L)	24.42 ± 7.81	28.81 ± 13.29	−2.042	0.044
IL-1β (pg/mL)	3.10 (2.00, 4.03)	3.00 (2.00, 3.70)	−0.381	0.703
IL-4 (pg/mL)	4.27 ± 1.29	4.93 ± 2.00	−1.982	0.050
IL-6 (pg/mL)	8.15 (2.95, 21.88)	14.40 (6.20, 33.78)	−2.049	0.040
IL-17 (pg/mL)	7.50 ± 2.22	8.37 ± 3.51	−1.475	0.143
IFN-γ (pg/mL)	7.20 (4.00, 10.65)	10.15 (6.83, 12.53)	−2.148	0.032
TNF-α (pg/mL)	3.00 (3.00, 4.13)	3.00 (3.00, 3.00)	−0.347	0.729
Lung consolidation, *n* (%)	22 (44%)	35 (67.3%)	5.617	0.018
Pleural effusion, *n* (%)	1 (2.0%)	13 (25%)	11.387	<0.001

Note. Data are presented as *n* (%), mean ± SD, or median (P25, P75).

**Table 3:** Proportion of SMPP across serum 25(OH)D concentration strata.

25(OH)D Stratum (ng/mL)	MMPP (*n* = 50)	SMPP (*n* = 52)
<12 (deficiency)	2 (13.3)	13 (86.7)
12–20 (insufficiency)	25 (50.0)	25 (50.0)
>20 (adequacy)	23 (62.2)	14 (37.8)

Note. Data are presented as *n* (%). The difference in the proportion of SMPP across the three strata was statistically significant (χ^2^ = 10.221, *p* = 0.006).

**Table 4:** Pulmonary function parameters between the two groups.

Parameter (% Predicted)	MMPP (*n* = 50)	SMPP (*n* = 52)	*t*	*p*
FVC	89.98 ± 13.58	88.09 ± 13.53	0.705	0.482
FEV_1_	92.47 ± 15.84	89.65 ± 14.98	0.923	0.358
FEV_1_/FVC	102.63 ± 6.98	102.10 ± 8.36	0.348	0.728
FEF_25_	83.11 ± 16.42	81.68 ± 18.75	0.410	0.683
FEF_50_	79.52 ± 19.20	79.44 ± 23.89	0.018	0.986
FEF_75_	72.14 ± 24.27	72.56 ± 31.68	−0.075	0.940

Note. Data are presented as mean ± SD. FVC, forced vital capacity; FEV_1_, forced expiratory volume in 1 s; FEF, forced expiratory flow.

**Table 5:** Distribution of pulmonary function classification between the two groups.

Pulmonary Function Category	MMPP (*n* = 50)	SMPP (*n* = 52)
NPF	22 (44.0)	21 (40.4)
SAD	15 (30.0)	15 (28.8)
VD	13 (26.0)	16 (30.8)

Note. Data are presented as *n* (%). The difference in the distribution of pulmonary function categories between the two groups was not statistically significant (χ^2^ = 0.294, *p* = 0.863). NPF, normal pulmonary function; SAD, small airway dysfunction; VD, ventilatory dysfunction.

**Table 6:** Multivariate logistic regression analysis of independent risk factors for SMPP.

Variable	β	SE	Wald	*p*	OR (95% CI)
25(OH)D < 12 ng/mL	2.426	1.018	5.675	0.017	11.31 (1.54–83.22)
CRP (mg/L)	0.032	0.015	4.752	0.029	1.033 (1.00–1.06)
LDH (U/L)	0.009	0.004	3.883	0.049	1.009 (1.00–1.01)
Fever duration (days)	0.440	0.146	9.118	0.003	1.552 (1.16–2.07)
Pleural effusion	3.734	1.222	9.337	0.002	41.86 (3.82–459.26)

## Data Availability

The data presented in this study are available on request from the corresponding author due to privacy.

## Abbreviations

The following abbreviations are used in this manuscript:

25(OH)D	25-hydroxyvitamin d
AST	Aspartate aminotransferase
AUC	Area under the curve
CI	Confidence interval
CRP	C-reactive protein
FEF	Forced expiratory flow
FEV_1_	Forced expiratory volume in 1 s
FVC	Forced vital capacity
IFN-γ	Interferon-gamma
IL	Interleukin
SAD	Small airway dysfunction
LDH	Lactate dehydrogenase
MPP	*Mycoplasma pneumoniae* pneumonia
NLR	Neutrophil-to-lymphocyte ratio
OR	Odds ratio
ROC	Receiver operating characteristic
SMPP	Severe *mycoplasma pneumoniae* pneumonia
TNF-α	Tumor necrosis factor-alpha
VD	Ventilatory dysfunction
VDR	Vitamin D receptor
